# ABCA7 deficiency causes neuronal dysregulation by altering mitochondrial lipid metabolism

**DOI:** 10.1038/s41380-023-02372-w

**Published:** 2023-12-22

**Authors:** Keiji Kawatani, Marie-Louise Holm, Skylar C. Starling, Yuka A. Martens, Jing Zhao, Wenyan Lu, Yingxue Ren, Zonghua Li, Peizhou Jiang, Yangying Jiang, Samantha K. Baker, Ni Wang, Bhaskar Roy, Tammee M. Parsons, Ralph B. Perkerson, Hanmei Bao, Xianlin Han, Guojun Bu, Takahisa Kanekiyo

**Affiliations:** 1 Department of Neuroscience, Mayo Clinic, Jacksonville, FL 32224 USA; 2 Center for Regenerative Biotherapeutics, Mayo Clinic, Jacksonville, FL 32224 USA; 3 Quantitative Health Sciences, Mayo Clinic, Jacksonville, FL 32224 USA; 4 Barshop Institute for Longevity and Aging Studies, University of Texas Health Science Center at San Antonio, San Antonio, TX 78229 USA; 5 Present Address: SciNeuro Pharmaceuticals, Rockville, MD 20850 USA; 6 Present Address: Division of Life Science, The Hong Kong University of Science and Technology, Clear Water Bay, Hong Kong, China

**Keywords:** Neuroscience, Stem cells

## Abstract

*ABCA7* loss-of-function variants are associated with increased risk of Alzheimer’s disease (AD). Using ABCA7 knockout human iPSC models generated with CRISPR/Cas9, we investigated the impacts of ABCA7 deficiency on neuronal metabolism and function. Lipidomics revealed that mitochondria-related phospholipids, such as phosphatidylglycerol and cardiolipin were reduced in the ABCA7-deficient iPSC-derived cortical organoids. Consistently, ABCA7 deficiency-induced alterations of mitochondrial morphology accompanied by reduced ATP synthase activity and exacerbated oxidative damage in the organoids. Furthermore, ABCA7-deficient iPSC-derived neurons showed compromised mitochondrial respiration and excess ROS generation, as well as enlarged mitochondrial morphology compared to the isogenic controls. ABCA7 deficiency also decreased spontaneous synaptic firing and network formation in iPSC-derived neurons, in which the effects were rescued by supplementation with phosphatidylglycerol or NAD^+^ precursor, nicotinamide mononucleotide. Importantly, effects of ABCA7 deficiency on mitochondria morphology and synapses were recapitulated in synaptosomes isolated from the brain of neuron-specific *Abca7* knockout mice. Together, our results provide evidence that ABCA7 loss-of-function contributes to AD risk by modulating mitochondria lipid metabolism.

## Introduction

Alzheimer’s disease (AD) is the most common neurodegenerative disease which causes dementia in the elderly. Approximately 6.2 million Americans over the age of 65 suffer from AD, with numbers expected to rise to 8 million by 2060 [1]. Accumulating evidence has shown the predominant contribution of genetic factors to AD onset and development [2]. Through genome-wide association studies (GWAS), variants in the ABCA7 gene encoding ATP-binding cassette (ABC) subfamily A member 7 have been identified as strong risk factors for late-onset AD [3–7]. Of note, *ABCA7* loss-of-function variants, including nonsense, frameshift, and canonical splice-site mutations, substantially increase the risk of AD [8]. The ABC transporter family is a superfamily of highly conserved integral membrane proteins that are responsible for transporting ions, peptides, proteins, and lipids across cellular membranes [9, 10]. Although ABCA7 is thought to primarily transport lipids and other lipophilic molecules [10], the mechanisms by which ABCA7 loss-of-function contributes to the pathogenesis of AD are not fully understood.

Recent advances in genome editing technologies, such as clustered regularly interspaced short palindromic repeats-associated protein 9 (CRISPR/Cas9) system, have contributed to clarifying the pathological mechanisms of neurodegenerative diseases using induced pluripotent stem cell (iPSC)-derived brain cell models [11]. Since ABCA7 is abundantly expressed in neurons [12], we developed isogenic ABCA7-deficient iPSC-derived neural models to investigate how ABCA7 loss-of-function impacts neuronal homeostasis. Here, we demonstrate that ABCA7 deficiency alters lipid metabolism and impairs mitochondrial properties in iPSC-derived cortical organoids and neurons, which is accompanied by synaptic dysregulations. Our findings provide new evidence that ABCA7 loss-of-function contributes to AD risk by disturbing neuronal mitochondria function. Therefore, targeting mitochondria-related lipid metabolism may be a promising avenue in developing AD therapies.

## Materials and methods

### Deletion of ABCA7 using CRISPR/Cas9 in human iPSC lines

Human iPSCs were generated from two healthy individuals (#1: MC0192; Female, 83 years old, *APOE* ε3/ε3 and #2: MC0117, Male, 71 years old, *APOE* ε3/ε3) and cultured in TeSR-E7 complete medium (Stemcell Technologies, Vancouver, Canada) on Matrigel (Corning, Corning, NY, USA) coated dish as reported previously [13]. The iPSCs were passaged with Dispase (Stemcell Technologies) every 6–9 days and subjected to treatment with ROCK inhibitor Y27632 (Sigma–Aldrich, Burlington, MA, USA) for the first 24 h. *ABCA7* deletion was performed in the iPSC lines through CRISPR/Cas9 using two gRNA-Cas9 plasmids (sgRNA-1: AGCCTCGTACCAGTTGAGGGAGG and sgRNA-2: CACTGCTGCAGAGACCCCGAGGG) by ALSTEM. Karyotyping of the iPSC clones was performed by Mayo Clinic Cancer Center in Rochester.

### Differentiation of iPSCs into cortical organoids

Cortical organoids were generated using the STEMdiff™ Cerebral Organoid Kit (Stemcell Technologies) according to the manufacturer’s instructions with minor modification [14, 15]. On day 0, iPSCs were dissociated into single-cell suspension with TrypLE Express (Thermo Fisher Scientific, Waltham, MA, USA) and cultured on U-bottom ultra-low-attachment 96-well plates (15,000 cells/well) in embryoid body (EB) formation media (medium A) supplemented with 10 μM Y27632. After the medium changes with EB formation media on day 2 and day 4, the iPSC-derived EBs were transferred onto 96-well low attachment plates on day 5 and cultured in the induction medium (medium B). On day 7, the EBs were transferred onto Matrigel-coated 6-well plates and cultured in the expansion medium (medium C + D) for cortical organoid formation for 3 days. On day 10, the culture medium was switched to the maturation medium (medium E). From day 10, the organoids were cultured on an orbital shaker followed by medium changes twice per week until day 60.

### Differentiation of iPSCs into NPCs and neurons

The differentiation from iPSC lines into NPCs was performed using the STEMdiff™ SMADi Neural Induction Kit (Stemcell Technologies) according to the manufacturer’s instructions with minor modification [13]. On day 0, dissociated iPSCs (3.0 × 10^6^ cells/well) were transferred onto 24-well AggreWell^TM^800 plates (Stemcell Technologies) for EB formation and cultured in STEMdiff™ SMADi Neural Induction Medium (Stemcell Technologies) supplemented with 10 μM Y27632 for 8 days. Half of the medium in each well was changed every 2 days. On day 8, the iPSC-derived EBs were transferred onto a Matrigel-coated 6-well plate and cultured in the induction medium supplemented with 10 μM Y27632 for another 5 days to induce neural rosette formation in the attached EB colonies. On day 15, the neural rosettes were carefully isolated from the surrounding EB-derived flat cells. The small clumps of isolated neural rosettes were transferred onto Matrigel-coated plates and cultured in the induction medium for additional 5–7 days. After removal of cells with non-rosette morphology, the neural rosettes were dissociated into single cells with TrypLE Express and replated on the Matrigel-coated 24-well plate and cultured in the induction medium supplemented with 10 μM Y27632 to initiate the differentiation into NPCs. The culture medium was switched to STEMdiff™ SMADi Neural Progenitor Medium (Stemcell Technologies) 1 day after the replating. For NPC expansion, the cells were passaged every 5–7 days with daily medium change, followed by cryopreservation with STEMdiff™ Neural Progenitor Freezing Medium (Stemcell Technologies). NPCs with passage number between 3 and 7 were cultured on the Matrigel-coated 24-well plate in STEMdiff™ Forebrain Neuron Differentiation Medium (Stemcell Technologies) to be differentiated into NPCs. After 5–7 days, NPCs were dissociated with TrypLE Express and cultured on Poly-L-ornithine (PLO, Sigma)/Laminin (Sigma)-coated plates in the maturation medium supplemented with 10 μM Y27632 to be differentiated into neurons. Half of the medium in each well was changed twice per week until used for experiments.

### Mice

*Abca7* floxed (*Abca7*^floxp/floxp^) mice were generated by Taconic Biosciences through a collaboration with the Cure Alzheimer’s Fund. Neuron-specific ABCA7 knockout (n*ABCA7*^−^^/^^−^) mice were generated by breeding the *Abca7*^floxp/floxp^ mice with Camk2a-Cre mice (EMMA: #01153, Munchen, Germany) [16, 17]. Male n*ABCA7*^−^^/^^−^ mice and control mice were analyzed at the age of 4 and 20 months. Frozen brain sections (20 µm thickness; coronal) were immunostained with the primary antibodies for ABCA7 (1:200, #25339-1-AP, Proteintech, Rosemont, IL, USA), NeuN (1:100, #MAB377, Millipore, Burlington, MA, USA), and GFAP (1:150, #ab279291, Abcam, Cambridge, UK) followed by incubations with the appropriate Alexa Fluor 488-, Alexa Fluor 568-, or Alexa Fluor 647-conjugated secondary antibodies (1:200, Thermo Fisher Scientific). Nuclei were then counterstained using DAPI (H-1500, Vector Laboratories, Newark, CA, USA). The images were taken with confocal laser scanning fluorescent microscopy (model LSM880 Invert, Carl Zeiss, Baden-Wurttemberg, Germany). Synaptosomes were isolated from the whole mouse brain using Syn-PERTM Synaptic Protein Extraction Reagent (Thermo Fisher Scientific) according to the manufacturer’s instructions. Briefly, the brain samples were homogenized in Syn-PER Reagent with Protease and Phosphatase inhibitor Cocktail (Thermo Fisher Scientific). The samples were centrifuged at 1200 *g* for 10 min and the remaining supernatants were transferred. The supernatants were then centrifuged at 15,000 *g* for 20 min, and the pellets (synaptosomes) were resuspended with Syn-PER Reagent with Protease and Phosphatase inhibitor Cocktail.

### Immunocytochemistry and staining for mitochondria and ER

The cells were immunostained with primary antibodies against TRA-1-60 (1:200, #ab16288, Abcam), NANOG (1:200, #D7364, Cell Signaling, Beverly, MA, USA), βIII-tubulin (1:500, #ab78078, Abcam; or 1:1000, #T2200, Sigma), MAP2 (1:200, #ab183830, Abcam), GFAP (1:100, #ab279291, Abcam), CTIP2 (1:50, #ab18465, Abcam), SATB2 (1:100, #ab34735, Abcam), SOX2 (1:250, #ab18102, Abcam), Nestin (1:500; #ab97959, Abcam), ABCA7 (1:500, #sc-377335, Santa Cruz Biotechnology, Dallas, TX, USA), and/or calreticulin (1:400, #12238, Cell Signaling) as previously described [18], followed by incubations with the appropriate Alexa Fluor 488- or Alexa Fluor 568-conjugated secondary antibodies (Thermo Fisher Scientific) and nuclei counterstaining with DAPI (300 nM, #D1306, Thermo Fisher Scientific). The level of reactive oxygen species (ROS) was analyzed as described previously [19]. Briefly, mitochondrial superoxide was quantified by staining with 10 μM MitoSOX red (Thermo Fisher Scientific) for 30 min. Mitochondria were stained with 200 nM MitoTracker Green FM (Thermo Fisher Scientific) for 30 min.

The MitoSOX/MitoTracker signal ratio was used to analyze the ROS level per mitochondria. The iPSC-derived neurons were incubated with 5 µM of NBD-PA_16:0-06:0 (1-palmitoyl-2-{6-[(7-nitro-2-1,3-benzoxadiazol-4-yl)amino]hexanoyl}-sn-glycero-3-phosphate) (#810173, Avanti Polar Lipids, Alabaster, AL, USA) and 1 µM of ER tracker (#E34250, Thermo Fisher Scientific) for 30 min. All images were taken with confocal laser scanning fluorescent microscopy (model LSM880 Invert, Carl Zeiss). The fluorescence of MitoSOX and MitoTracker were measured using SpectraMax M5 (Molecular Devices, San Jose, CA, USA). The mean fluorescence intensity of ER tracker or NBD PA co-localized with ER tracker were measured with ImageJ.

### Transmission electron microscopy (TEM)

The iPSC-derived cortical organoids, neurons, and mouse synaptosomes were fixed with PBS containing 2% glutaraldehyde and 2% PFA, followed by post-fixation with 1% OsO_4._ The samples were stained with 1% uranyl acetate in 50% ethanol for 30 min, then sequentially dehydrated with increased concentrations of ethanol (70%, 80%, 95%, and 100%) and at last 100% propylene oxide for 10 min each time. After the dehydration, samples were infiltrated with the 1:1 mixture of 100% propylene oxide and Epon 812 (Polysciences, Warrington, PA, USA) overnight at room temperature, then embedded in Epon 812, followed by polymerization at 60 °C for 2 days. Ultrathin sections (100 nm) were sliced from the Epon 812 embedded samples by Leica Ultramicrotome (UC7), and subsequently counterstained with uranyl acetate and lead citrate. The sections were imaged using JEM-1400 Flash Transmission Electron Microscopy (JEOL). Mitochondrial morphology (length, width, perimeter, and circularity) was measured with ImageJ [20].

### qRT-PCR

Total RNA was isolated from iPSCs, iPSC-derived cortical organoids, and mouse brains with RNeasy® Mini Kit (Qiagen, Hilden, Germany). Reverse transcription was performed using SuperScript® III First-Strand Synthesis System (Thermo Fisher Scientific). The qRT-PCR was performed using SsoAdvanced Universal SYBR® Green Supermix (Bio-Rad, Hercules, CA, USA) or TaqMan™ Gene Expression Master Mix (Thermo Fisher Scientific) using Quantstudio 7 (Thermo Fisher Scientific). Gene-expression levels were normalized to *GAPDH* or *Gapdh* expression. All primers were purchased from IDT, Inc. and Thermo Fisher Scientific: (Supplementary Table 1).

### Western blotting

Samples were lysed with RIPA Buffer (Boston BioProducts, Inc., Milford, MA, USA) containing Protease and Phosphatase Inhibitor Cocktail (Thermo Fisher Scientific) and subjected to Western blotting [14, 15]. Anti-ATP5A (1:500, #ab110413, Abcam), anti-β-actin (1:3000, #A2228, Millipore), anti-caspase-3 (1:1000, #9662, Cell Signaling Technology), anti-cleaved caspase-3 (1:1000, #9662, Cell Signaling Technology), anti-ABCA7 (1:500, #sc-377335, Santa Cruz Biotechnology), anti-ABCA7 (1:500, #MABI 97-17, MAB Institute, Kanagawa, Japan), anti-βIII-tubulin (1:1000, #ab78078, Abcam), anti-PSD95 (1:1000, #ab18258, Abcam), anti-SNAP25 (1:1000, #PA5-85396, Thermo Fisher Scientific), anti-TIM23 (1:100, #sc-514463, Santa Cruz Biotechnology), anti-MFN2 (1:1000, #ab56889, Abcam), ant-DRP1 (1:500, #ab184247, Abcam), and anti-FIS1 (1:500, #PA5-22142, Thermo Fisher Scientific) were used as primary antibodies. The following secondary antibodies were used: IRDye®800CW Goat anti-Mouse and anti-Rat (1:4000, #926-32210 and #926-32219, LI-COR Biosciences, Lincoln, NE) and IRDye®680RD Goat anti-Rabbit (1:4000, #926-32211, LI-COR Biosciences). Immunoreactivities were detected and quantified using Odyssey Infrared Imaging System (LI-COR Biosciences).

### Measurement of ATP synthase activity and advanced oxidation protein product (AOPP) levels

ATP synthase activity was measured using ATP Synthase Enzyme Activity Microplate Assay Kit (Abcam) according to the manufacturer’s instructions. The absorbance of the product was measured at 340 nm using Synergy^TM^ HT Microplate Reader (BioTek, Winooski, VT, USA). AOPP levels were measured using the AOPP Assay Kit (Abcam) according to the manufacturer’s instructions. The absorbance of the product was measured at 340 nm using Synergy^TM^ HT Microplate Reader (BioTek).

### Microelectrode array (MEA) electrophysiology

The iPSC-derived NPCs were cultured on the Matrigel-coated 24-well plate with STEMdiff™ Forebrain Neuron Differentiation Medium (Stemcell Technologies) for 5–7 days. The NPCs dissociated with TrypLE Express were transferred onto Poly-ethyleneimine (PEI, Sigma)/Laminin (Sigma)-coated MEA 24-well Plate-eco (1 × 10^5^ cells/well) and to differentiated into neurons in STEMdiff™ Forebrain Neuron Maturation (Stemcell Technologies) supplemented with 10 μM Y27632. Spontaneous spikes were recorded for 10 min with MED64 Presto (Alpha Med Scientific, Osaka, Japan) weekly from 2 weeks after the differentiation. A spike was detected when the recorded signal exceeded a threshold of ±5 σ, where σ was the standard deviation of the baseline noise during quiescent periods. Burst firing was detected as the group of spikes with less than the average inter-spike interval (ISI), and the burst duration time was decided using Poisson surprise [21]. The recorded spikes and burst firing were analyzed with MEA Symphony and Burst Analysis Table software (Alpha Med Scientific).

### Oxygen consumption rate (OCR) measurement

iPSCs, iPSC-derived NPCs, or neurons were plated on Matrigel or Poly-L-ornithine (PLO, Sigma)/Laminin (Sigma)-coated Seahorse XF96 Cell Culture Microplate (Agilent, Santa Clara, CA, USA). The iPSC-derived neurons were cultured for 6 weeks, and then Oxygen Consumption Rate (OCR) values were measured using Seahorse XFe96 Extracellular Flux Analyzer (Agilent) in accordance with the manufacturer’s instructions in the XF Cell Mito Stress Test Kit (Agilent). OCR at the iPSC and NPC stages, cells were measured 1 day after the plating. Oligomycin (1.5 μM for iPSCs and 2.5 µM for NPCs/neurons), FCCP (0.5 μM for iPSCs and 1 μM for NPCs/neurons), and rotenone/antimycin A (0.5 μM) were used. After OCR was measured, the cells were fixed with PBS containing 4% PFA, stained with DAPI (Thermo Fisher Scientific), and the fluorescence intensity was measured using SpectraMax M5 (Molecular Devices) to assess the cell density. OCR in each well was normalized by the cell density.

### Administrations with nicotinamide mononucleotide (NMN), phosphatidylglycerol (PG), cardiolipin (CL), and phosphatidic acid (PA)

NMN (#S5259, Selleck Chemicals LLC, Houston, TX, USA) was dissolved in deuterium-depleted water (DDW) at 100 mM as a stock solution. PG_18;2-18:2 (1,2-dilinoleoyl-sn-glycero-3-phospho-(1′-rac-glycerol); #840485, Avanti Polar Lipids), CL (bovine heart-derived cardiolipin (#840012, Avanti Polar Lipids)), and PA (chicken egg-derived L-α-phosphatidic acid; #840101, Avanti Polar Lipids) were dissolved in dimethyl sulfoxide (DMSO) at 50, 25, and 25 mM, respectively, as a stock solution. In all experiments, culture medium containing DDW or DMSO (0.1–0.2 %) was used as respective controls. The concentrations of NMN, PG, CL, and PA were selected based on their relevance in previous reports [22–25].

### RNA-seq

RNA-seq was performed at Mayo Clinic sequencing core using Illumina HiSeq 4000 as described previously [14, 15]. Reads were aligned to the human reference genome hg38. Mayo Clinic RNA-Seq analytic pipeline (MAP-RSeq Version 3.1.3) was used [26] to generate raw gene read counts and sequencing quality control metrics. Raw gene counts were corrected for gene length differences, GC bias, and global technical variations through Conditional Quantile Normalization (CQN) [27]. Based on the bi-modal distribution of the CQN normalized and log2-transformed reads per kb per million (RPKM) gene-expression values, genes with an average of log2 RPKM ≥ 0 in at least one genotype group were included in the analysis. Using this threshold, 17,912 genes were identified for downstream analysis. PCA and differentially expressed genes (DEG) analysis were performed using Partek Genomics Suite (Partek Inc., St. Louis, MO, USA). An ANOVA model was used to compare gene expressions between *ABCA7*^−^^*/*^^−^ iPSC-derived cortical organoids and isogenic controls while adjusting for the confounding effects of RNA Integrity Numbers (RIN). Volcano plots of differentially expressed genes were generated using R version 3.4.1. Pathway analyses of differentially expressed genes were performed using Ingenuity Pathway Analysis (QIAGEN) [28].

### Lipidomics

Shotgun lipidomic analysis was conducted through the multidimensional mass spectrometry system as described previously [15]. Samples were homogenized with Precellys Lysing Kit (Bertin Instruments, Montigny-le-Bretonneux, France) using Cryolys Evolution homogenizer (Bertin Instruments) and subjected to lipid extraction through modified Bligh and Dyer procedure [29]. Each lipid extract was reconstituted in organic solvent composed of chloroform and methanol (1:1 volume ratio, 400 µl/mg protein) and diluted to a final total lipid concentration of ~500 fmol/µl. Mass spectrometric analysis was performed on a triple quadrupole mass spectrometer (TSQ Altis, Thermo Fisher Scientific) and a Q Exactive mass spectrometer (Thermo Fisher Scientific), which were connected with an automated nanospray device (TriVersa NanoMate, Advion Inc, Ithaca, NY, USA) [30]. Amounts of lipid species were normalized to the protein concentrations [31, 32]. For WGCNA in the lipidomics data, a power of 12, a minimum module size of 10 lipids, and a minimum height for merging modules of 0.25 were employed to construct an unsigned network. Modules were annotated through R package anRichment. Hub lipids were defined based on the connectivity in each module.

### Statistical analysis

All statistical analyses were performed using EZR software and GraphPad Prism 9 except for RNA-seq and lipidomics. Comparisons were performed using two-tailed student’s *t*-test, paired *t*-test, Tukey–Kramer post hoc analysis of Two-way ANOVA, or repeated measures two-way ANOVA. *P* values < 0.05 were considered statistically significant.

## Results

### ABCA7 deficiency leads to synaptic protein reductions in iPSC-derived cortical organoids

**Fig. 1:**
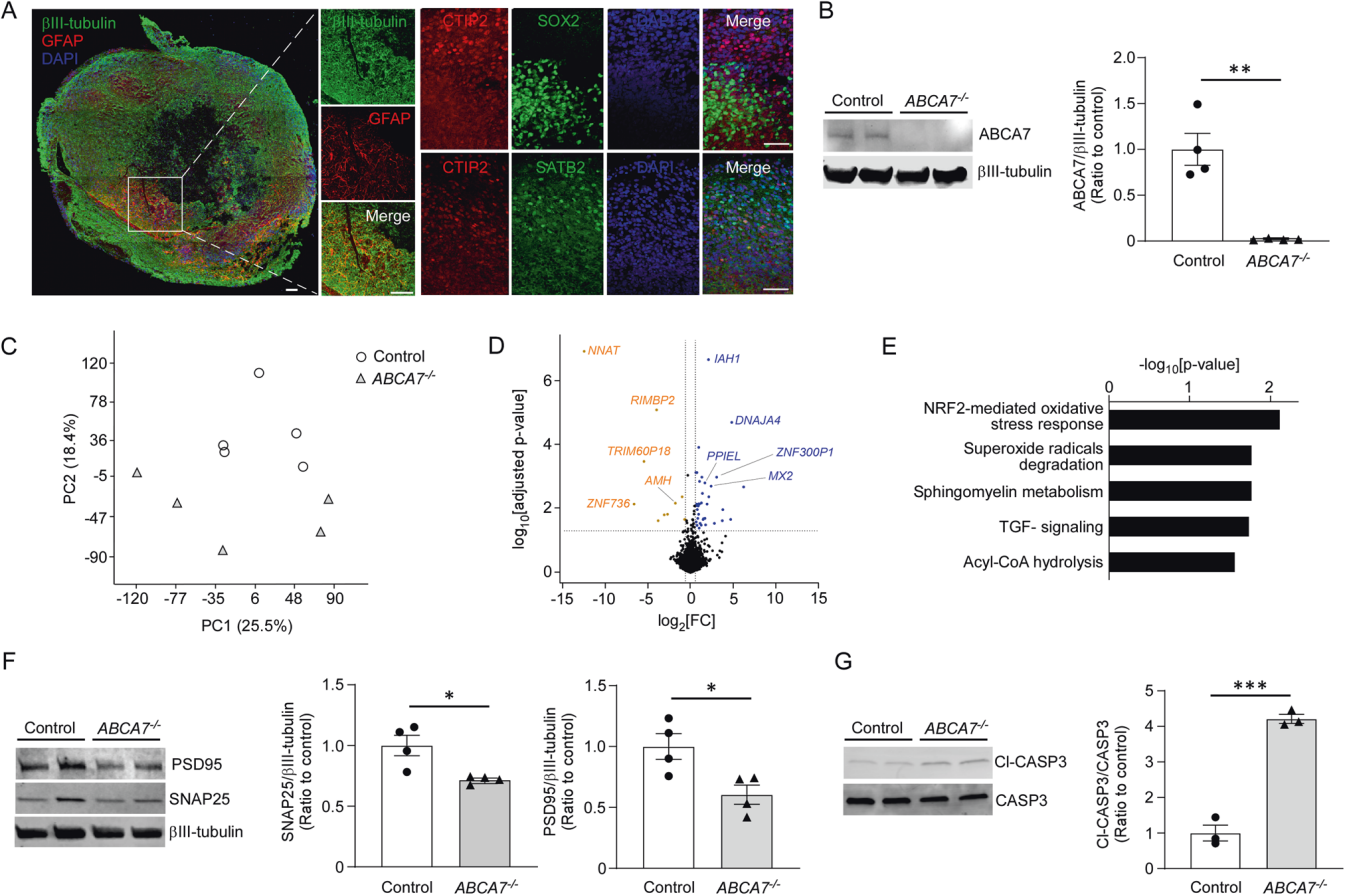
Characterization and synaptic loss in *ABCA7*^−^^/^^−^ iPSC-derived cortical organoids. Isogenic control and *ABCA7*^−^^/^^−^ iPSCs (#1) were differentiated into cortical organoids and analyzed at Day 60 of differentiation. **A** Representative images of the ventricular zone (VZ)-like structure (βIII-Tubulin, neuronal marker; SOX2, neural progenitor cell marker; GFAP, astrocyte maker; CTIP2, deep cortical layer marker; and SATB2 superficial cortical layer marker) in cerebral organoids. Nuclei were stained with DAPI. Scale bars: 100 µm. **B** ABCA7 levels in the iPSC organoids were analyzed by Western blotting and normalized by βIII-tubulin levels (*n* = 4 technical replicates/line). **C**–**E** Bulk RNA-sequencing was performed in the iPSC-derived cortical organoids. PCA plot (**C**), volcano plot (**D**), and the top 5 pathways enriched by DEGs (**E**) are shown (*n* = 6 technical replicates/line). The step-up adjustment was based on the Benjamin-Hochberg procedure. **F** Levels of presynaptic protein (SNAP25) and postsynaptic protein (PSD95) in the iPSC organoids were analyzed by Western blotting and normalized by βIII-tubulin levels (*n* = 4 clones/line). **G** Levels of cleaved caspase 3 and full-length caspase 3 in the iPSC organoids were analyzed by Western blotting and their ratio was plotted (*n* = 3 technical replicates/line). Three cortical organoids cultured in an identical dish were lysed together and analyzed as one sample. Data represents mean ± SEM. *p < 0.05, **p < 0.01 by two-tailed student’s *t*-test.

To model ABCA7 loss-of-function in human cells, we generated two isogenic ABCA7 knockout iPSC lines (*ABCA7*^−^^*/*^^−^) by using CRISPR/Cas9. The *ABCA7*^−^^*/*^^−^ iPSC lines exhibited typical morphology, expressed pluripotent markers, and had normal karyotypes (Supplementary Fig. 1). The iPSCs were differentiated into cortical organoids and analyzed 60 days after the differentiation. The iPSC-derived cortical organoids were mainly composed of βIII-tubulin-positive neurons and GFAP-positive astrocytes. Deep cortical layer marker CTIP2 aligned with SOX2-positive neural progenitor cells as well as superficial cortical layer marker SATB2 were also detected (Fig. 1A). When isogenic control and *ABCA7*^−^^*/*^^−^ iPSC-derived cortical organoids (Fig. 1B) were subjected to bulk RNA-sequencing (RNA-seq), principal component analysis (PCA) plot revealed two distinct clusters associating with each group with 25.5% variance captured by PC1 and 18.4% variance captured by PC2 (Fig. 1C). Among 65 DEGs with step-up *p*-value less than 0.05, 15 genes were downregulated in the *ABCA7*^−^^*/*^^−^ iPSC-derived organoids compared to the isogenic controls, which include *NNAT* coding neuronatin and *RIMB2* coding RIMS binding protein 2. The upregulated 50 genes included *IAH1* coding isoamyl acetate hydrolyzing esterase 1 and *DNAJA4* coding DnaJ Heat Shock Protein Family (Hsp40) Member A4 (Fig. 1D). We also confirmed the results using qRT-PCR (Supplementary Fig. 2A). NNAT is involved in neuronal growth and maintenance of the structure of the nervous system and RIMBP2 mediates synaptic transmission [33–35]. Furthermore, the gene ontology analysis identified “NRF2-mediated Oxidative Stress Response” and “Superoxide Radical degradation” as the top 2-ranked canonical pathways (Fig. 1E). Consistent with the results from RNA-seq, we found the reductions of presynaptic protein SNAP25 and postsynaptic protein PSD95 in *ABCA7*^−^^*/*^^−^ iPSC-derived organoids (Fig. 1F). Furthermore, the ratio of caspase 3 and cleaved caspase 3 was increased in *ABCA7*^−^^/^^−^ iPSC-derived organoids (Fig. 1G). Since caspase 3 activation mediates oxidative stress-induced apoptosis [36], these results indicate that ABCA7 deficiency induces oxidative stress and apoptosis accompanied with the impairment of proper synaptic formation in iPSC-derived cortical organoids. We also observed increased mRNA expression levels of apoptosis-related genes, including *APAF1*, *BAK1*, and *XIAP* in *ABCA7*^−^^*/*^^−^ cortical organoids compared to the isogenic controls (Supplementary Fig. 2B).

### ABCA7 deficiency alters lipid profiles and leads to mitochondrial dysfunction in iPSC-derived cortical organoids

**Fig. 2:**
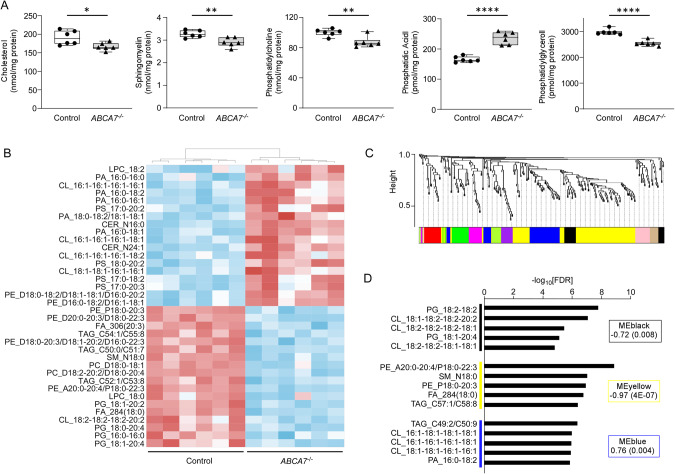
Altered lipid profile in *ABCA7*^−^^/^^−^ iPSC-derived cortical organoids. Lipidomics was performed in the cortical organoids derived from isogenic control and *ABCA7*^−^^/^^−^ iPSCs (#1). Three iPSC organoids cultured in an identical dish were lysed together and analyzed as one sample (*n* =  6 technical replicates/line). All lipid concentrations were normalized to total protein levels. **A** Major lipid species with significant differences are shown. Data represent mean ± SEM. *p < 0.05, **p < 0.01, ****p < 0.0001 by two-tailed student’s *t*-test. **B** Heat map of lipid subspecies altered by ABCA7 deficit is shown. **C** The correlation between lipid module eigengenes and ABCA7 deficit is shown. **D** The black, yellow, and blue modules showed a correlation with *p* < 0.05. The FDR of the top 5 hub lipids was visualized.

To explore the effects of ABCA7 loss-of-function on lipid metabolism, lipidomics analysis was conducted on cell lysates of cortical organoids derived from isogenic control and *ABCA7*^−^^*/*^^−^ iPSCs 60 days after differentiation. Among non-polar lipids, we found that free cholesterol levels were decreased in *ABCA7*^−^^*/*^^−^ iPSC-derived organoids. However, levels of total cholesterol ester (CE), fatty acids (FA) and triacylglycerol (TAG) were not affected. In addition, lower total levels of sphingomyelin (SM), phosphatidylcholine (PC) and phosphatidylglycerol (PG), but higher phosphatidic acid (PA), were detected in the *ABCA7*^−^^*/*^^−^ iPSC-derived cortical organoids compared to the isogenic controls (Fig. 2A). Since synapses are enriched in cholesterol, SM and PS [37], the reduced synaptic protein levels in *ABCA7*^−^^*/*^^−^ iPSC-derived organoids may be associated with disorganizations of synaptic lipid membranes. The lipidomes, particularly those belonging to PA, PG, and cardiolipin (CL), were broadly altered between isogenic control and *ABCA7*^−^^/^^−^ iPSC-derived organoids (Fig. 2B). The Weighted Gene Co-expression Network Analysis (WGCNA) of the lipidomics dataset (Fig. 2C) identified three modules that were changed in *ABCA7*^−^^/^^−^ cortical organoids compared to controls: MEblack (−0.72; *p* = 0.02), MEyellow (−0.97; *p* = 4E-07) and MEblue (0.76; *p* = 0.004) (Fig. 2D). MEblack contains PG and CL with C18:2 acyl chain as the top 5 ranked hub lipids, which were reduced in *ABCA7*^−^^/^^−^ cortical organoids. While CL with C16:1 or C18:1 acyl chain and PA_C16:0-C18:2 are identified in MEblue, those lipids were increased in *ABCA7*^−^^/^^−^ cortical organoids. PA is synthesized in the ER and transported to the mitochondrial outer membrane. A portion of PA is further transported to the inner membrane, where it is converted to PG and finally CL [38]. Since CL with C18:2 acyl chain is a major component of the mitochondrial inner membrane, those results suggest that ABCA7 deficiency may compromise the transport of PA to mitochondria, resulting in the disturbances of synthesis for PG and CL. In addition, MEyellow was relatively enriched with PE subspecies. As PE is also synthesized from PA-derived phosphatidylserine (PS) in mitochondria [39], ABCA7 loss-of-function may specifically influence the lipid components of mitochondria.

In addition, electron microscopy revealed inflated mitochondria morphologies with vague membrane structures in *ABCA7*^−^^/^^−^ iPSC-derived cortical organoids compared to the isogenic controls (Supplementary Fig. 3A). We also found lower protein levels of catalytic subunit of the ATP synthase complex (Supplementary Fig. 3B) and lower ATP synthase activity in *ABCA7*^−^^/^^−^ iPSC-derived organoids compared to controls (Supplementary Fig. 3C). Whereas ATP synthase activity is bidirectionally associated with production of reactive oxygen species (ROS) [40], advanced oxidation protein product (AOPP) level was significantly higher in *ABCA7*^−^^/^^−^ iPSC-derived organoids compared to controls (Supplementary Fig. 3D). Since the mitochondria is a central organelle for ATP synthesis and ROS production, these results indicate that ABCA7 deficiency disturbs mitochondria lipid homeostasis and compromises mitochondria functions in iPSC-derived cortical organoids. There were no differences in mRNA expression levels of mitochondria-related genes between isogenic control and *ABCA7*^−^^/^^−^ iPSC-derived organoids in RNA-seq data and qRT-PCR validation (Supplementary Fig. 4).

### ABCA7 deficiency impairs mitochondrial respiration and alters mitochondrial morphology in the iPSC-derived neurons

**Fig. 3:**
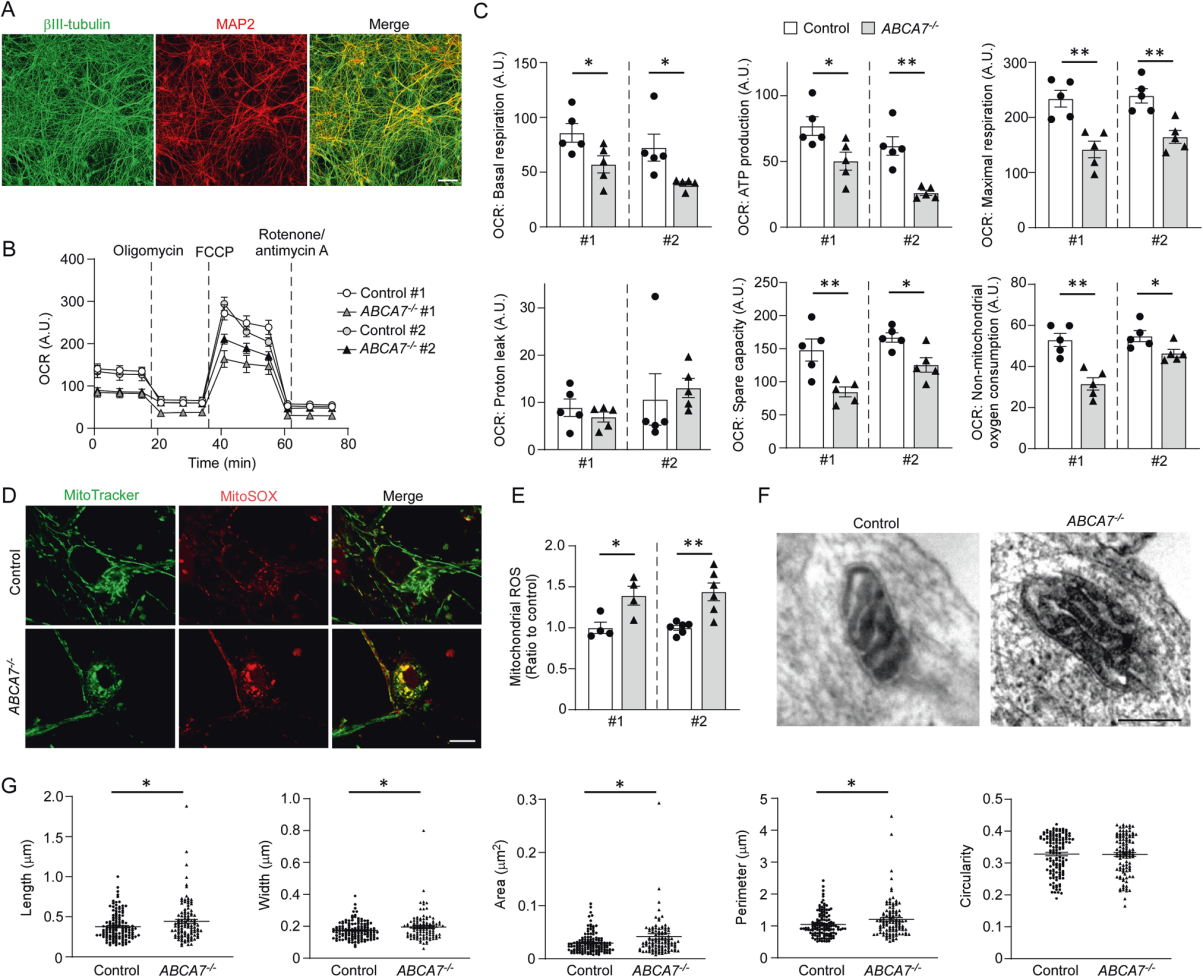
Impaired mitochondrial respiration and altered morphology in *ABCA7*^−^^*/*^^−^ iPSC-derived neurons. **A** Immunocytochemical staining of iPSC-derived neurons 6 weeks after differentiation from NPCs for neuron markers with βIII-tubulin and MAP2. Nuclei were stained with DAPI. Scale bar: 100 µm. **B**, **C** Mitochondrial respiration in neurons derived from isogenic control and *ABCA7*^−^^*/*^^−^ iPSCs (#1 and #2) were measured by Mito Stress Test Kit through Seahorse XFe96 Extracellular Flux Analyzer 6 weeks after differentiation. The OCR measurements were normalized to cell density determined by nuclear DAPI staining in each well (*n* = 5 technical replicates/line). A.U., arbitrary unit. **D**, **E** The iPSC-derived neurons were stained for MitoSOX and MitoTracker. Mitochondrial ROS was assessed as relative MitoSOX/MitoTracker ratio (*n* = 4–6 technical replicates/line). Scale bars: 200 nm. **F**, **G** Representative electron microscope images of mitochondria in the iPSC-derived neurons are shown. The length, width, area, perimeter, and circularity of mitochondria in neurons derived from isogenic control and *ABCA7*^−^^*/*^^−^ iPSCs (#1) were measured with ImageJ. (Control: *n* = 134 mitochondria from 3 clones, *ABCA7*^−^^/^^−^: *n* = 107 mitochondria from 3 technical replicates). Scale bar: 100 nm. Data represents mean ± SEM. *p < 0.05, **p < 0.01 by two-tailed student’s *t*-test.

In order to further investigate the role of ABCA7 in neuronal functions, isogenic control and *ABCA7*^−^^/^^−^ iPSC-derived NPCs were differentiated into neurons (Fig. 3A). Immunocytochemistry revealed that ABCA7 is partially co-localized with the notable ER marker, calreticulin, in iPSC-derived neurons (Supplementary Fig. 5A). Moreover, we found that exogenously added NBD-PA_16:0-16:0 accumulated within the ER of *ABCA7*^−^^/^^−^ iPSC-derived neurons at much higher levels than in controls (Supplementary Fig. 5B–D). These results suggest that ABCA7 deficiency prevents the proper distribution of lipid from the ER to other organelles in iPSC-derived neurons. Next, mitochondrial respiration was assessed through oxygen consumption rate (OCR) measurements using Seahorse XFe96 Extracellular Flux Analyzer (Fig. 3B). Consistent with the results from iPSC-derived cortical organoids, major mitochondrial respiration parameters, including basal respiration, ATP-linked respiration, maximal respiration, and spare respiratory capacity, were reduced in *ABCA7*^−^^/^^−^ iPSC-derived neurons compared to those from controls (Fig. 3C). However, there were no evident differences in mitochondrial respiration parameters at iPSC or NPC stages, although we observed reductions of proton leakage in *ABCA7*^−^^*/*^^−^ iPSC-derived NPCs (Supplementary Fig. 6). We also found that ABCA7 deficiency increased mitochondrial ROS accumulation in iPSC-derived neurons (Fig. 3D, E). When the mitochondrial morphology of iPSC-derived neurons was quantitatively evaluated, the length, width, perimeter, and area of mitochondria in the *ABCA7*^−^^/^^−^ iPSC-derived neurons were larger than those of the controls (Fig. 3F, G). These results indicate that proper mitochondrial dynamics, ATP production, and ROS generation were disturbed in neurons in the absence of ABCA7.

### ABCA7 deficiency suppresses spontaneous electrical firing and burst firing in the iPSC-derived neurons

**Fig. 4:**
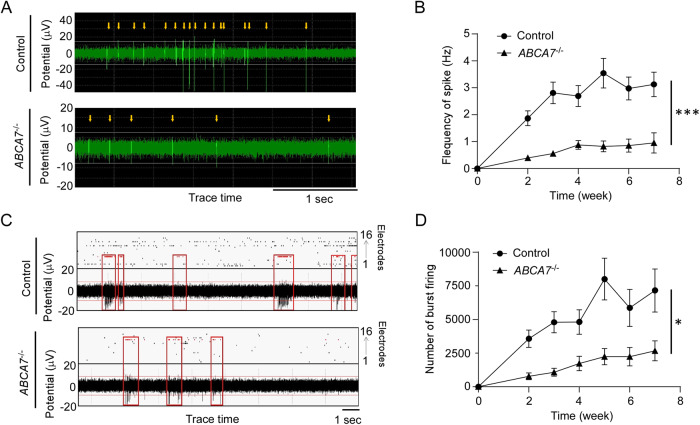
Impaired spontaneous electrical activity and synaptic network formation in *ABCA7*^−^^*/*^^−^ iPSC-derived neurons. **A** Spontaneous firing patterns in neurons derived from isogenic control and *ABCA7*^−^^*/*^^−^ iPSCs were measured by MED64 PRESTO 7 weeks after differentiation from NPCs. Each spike is indicated with a yellow arrow. **B** The frequency of spontaneous firing was monitored in the iPSC neurons for 7 weeks after differentiation (*n* = 9–10 technical replicates from 2 isogenic lines (#1 and #2)/group). **C** Extracellular recordings of spontaneous firing including burst firing (red rectangles) in the iPSC-derived neurons were measured by MED64 PRESTO 7 weeks after differentiation. **D** The incidence of burst firing was monitored in the iPSC neurons for 7 weeks after differentiation (*n* = 9–10 technical replicates from 2 isogenic lines (#1 and #2)/group). Data represents mean ± SEM. *p < 0.05, ***p < 0.001 by two-tailed student’s *t*-test at week 7.

To investigate how ABCA7 deficit influences neuronal activity, the electrophysiological activities of synaptic networks in iPSC-derived neurons were measured using microelectrode array (MEA) systems [41]. While the frequency of spontaneous spikes increased in the control iPSC-derived neurons in a time-dependent manner, ABCA7 deficiency substantially decreased the spike frequency (Fig. 4A, B). Next, synaptic network maturation was assessed by measuring the number of burst firings in the iPSC-derived neurons. The number of burst firings increased in control iPSC-derived neurons throughout maturation. However, fewer burst firings were detected in *ABCA7*^−^^/^^−^ iPSC-derived neurons than in controls when monitored for 7 weeks (Fig. 4C, D). Our findings provide evidence that ABCA7 deficiency suppresses spontaneous electrical activity and maturation of synaptic networks in iPSC-derived neurons.

### Administration of phosphatidylglycerol (PG) and nicotinamide mononucleotide (NMN) restores mitochondrial respiration and synaptic function in ABCA7-deficient iPSC-derived neurons

**Fig. 5:**
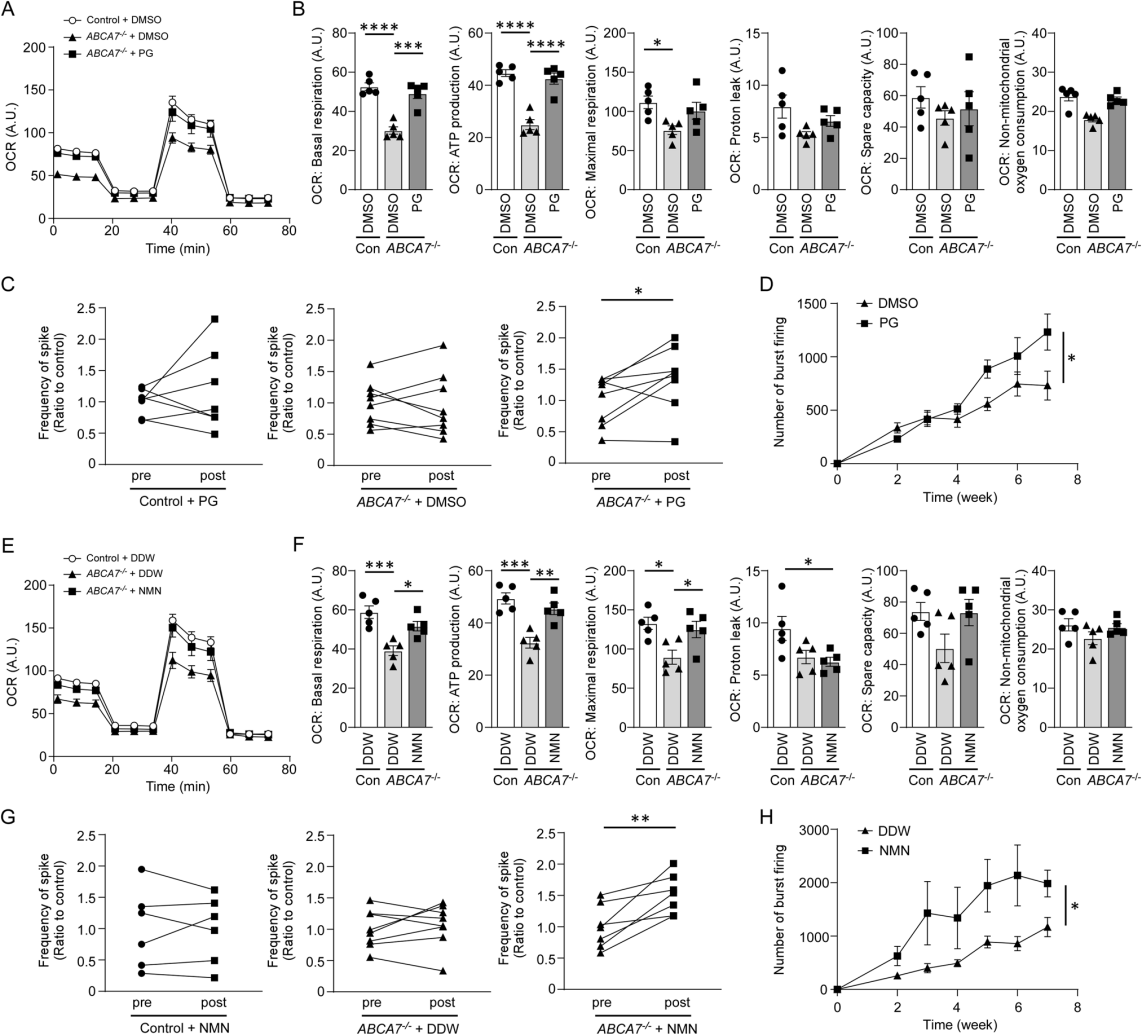
Restored mitochondrial respiration and synaptic function by supplementation with NMN or PG in *ABCA7*^−^^*/*^^−^ iPSC-derived neurons. **A**, **B**, **E**, **F** Mitochondrial respiration in neurons derived from isogenic control and *ABCA7*^−^^*/*^^−^ iPSCs (#1) was measured by Mito Stress Test Kit through Seahorse XFe96 Extracellular Flux Analyzer with PG_18:2-18:2 (**A**, **B**; 50 µM) or NMN (**E**, **F**; 100 µM) administration for 1 day 7 weeks after differentiation from NPCs. DMSO or DDW was used as a control, respectively. The OCR measurements were normalized with cell density determined by nuclear DNA staining with DAPI (*n* = 5–7 technical replicates/group). A.U., arbitrary unit. **C**, **G** Frequency of spontaneous firing was measured in the control and *ABCA7*^−^^*/*^^−^neurons (#1) before and after administration with PG_18:2-18:2 (**C**; 50 µM), NMN (**G**; 100 µM), or respective controls (*n* = 7–8 technical replicates/group). Data were normalized to those before adding the compound. **D**, **H** The *ABCA7*^−^^*/*^^−^neurons (#1) were treated with PG_18:2-18:2 (**D**; 50 µM), NMN (**H**; 100 µM), or respective controls 1 week after the differentiation into neurons for 7 weeks and number of burst firings represents were monitored (*n* = 4–5 technical replicates/group). Data represents mean ± SEM. *p < 0.05, **p < 0.01, ***p < 0.001, ****p < 0.0001 by paired *t*-tests (**C**, **G**), Tukey–Kramer post hoc analysis of Two-way ANOVA (**B**, **F**) or repeated measures two-way ANOVA (**D**, **H**).

Because lipidomics revealed significant reduction of PG_18:2-18:2 in *ABCA7*^−^^/^^−^ iPSC-derived cortical organoids (Fig. 2D), we investigated whether the PG supplementation can alleviate mitochondrial and synaptic dysfunctions in *ABCA7*^−^^/^^−^ iPSC-derived neurons. We found that mitochondrial respirations, specifically basal respiration, and ATP-linked production, were restored in *ABCA7*^−^^/^^−^ iPSC-derived neurons after PG_18:2-18:2 administration for 24 h (Fig. 5A, B, and Supplementary Fig. 7A, B). In addition, CL supplementation also ameliorated basal respiration and ATP-linked production, while PA supplementation did not influence mitochondrial respiration (Supplementary Fig. 7C–F). Next, the effects of PG_18:2-18:2 administration on neuron activity were assessed through MEA in the isogenic control and *ABCA7*^−^^/^^−^ iPSC-derived neurons. We observed a notable increase of spontaneous electrical activity in *ABCA7*^−^^*/*^^−^ iPSC-derived neurons 24 h after the administration with PG. No effect of PG administration was detected in control iPSC-derived neurons (Fig. 5C). Furthermore, the impaired burst firing in *ABCA7*^−^^*/*^^−^ iPSC-derived neurons was ameliorated by PG_18:2-18:2 supplementation starting 1 week after the differentiation (Fig. 5D). To further investigate whether mitochondrial alternation is the causative mechanism of synaptic dysfunction in *ABCA7*^−^^/^^−^ iPSC-derived neurons, we treated the neurons with the nicotinamide adenine dinucleotide (NAD^+^) precursor, NMN [42]. NMN administration for 24 h substantially ameliorated mitochondrial respiration parameters such as basal respiration, ATP-linked production, and maximal respiration in *ABCA7*^−^^/^^−^ iPSC-derived neurons (Fig. 5E, F). Furthermore, NMN had a rescuing effect on spontaneous firing (Fig. 5G) and burst firing (Fig. 5H) in *ABCA7*^−^^*/*^^−^ iPSC-derived neurons. Overall, our results support that ABCA7 deficiency disturbs mitochondria lipid homeostasis and mitochondrial respiration, thereby diminishing synaptic activity and network formation in iPSC-derived neurons.

### Neuronal *Abca7* deletion alters mitochondria morphology and reduces synaptic proteins in synaptosomes

**Fig. 6:**
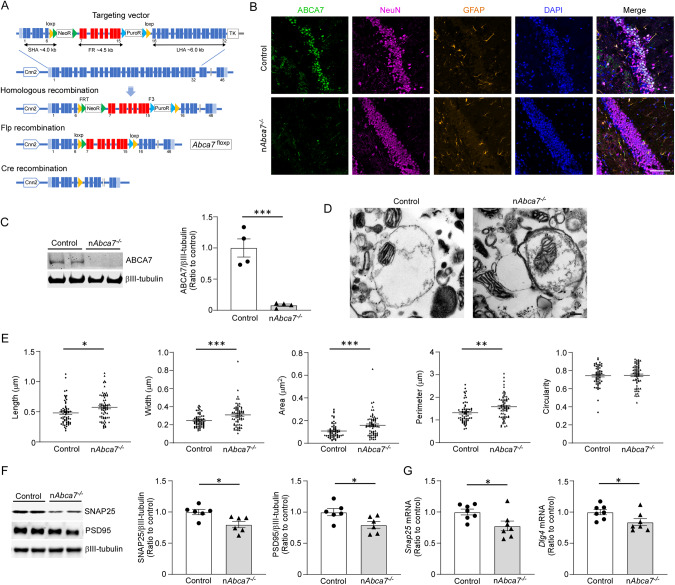
Mitochondrial morphological changes and synaptic loss in synaptosomes extracted from neuron-specific *Abca7* knockout mice. **A** The schematic process of *Abca7*^floxp^ allele generation is shown. **B** Neuron-specific *Abca7* knockout (n*Abca7*^−/−^) mice and control mice were generated by crossing *Abca7*^floxp/floxp^ mice with Camk2a-Cre^+/^^−^ mice. Representative images of immunostaining for ABCA7, NeuN, and GFAP in the hippocampus from the mice are shown. Nuclei were stained with DAPI. Scale bars: 100 µm. **C** Synaptosomes were extracted from the mouse brains at the age of 20 months and ABCA7 levels were assessed by Western blotting. Data were normalized to those of βIII-tubulin. (*N* = 4 male mice/group). **D**, **E** Electron microscope images of synaptosomes derived from the mice. The length, width, area, and perimeter of mitochondria were measured with ImageJ. Scale bar: 200 nm. (Control: *n* = 62 from 3 male mice, n*ABCA7*^−/−^: *n* = 69 from 3 male mice). **F** Amounts of presynaptic protein (SNAP25) and postsynaptic protein (PSD95) in the synaptosomes were assessed by Western blotting. Data were normalized to those of control lines. (*N* = 6 male mice/group). **G** Expressions of *Snap25 and Dlg4* mRNA in the cortex of the mouse brain were measured by qRT-PCR and normalized by *Gapdh* mRNA level (*N* = 7 male mice/group). Data represents mean ± SEM. *p < 0.05, **p < 0.01, ***p < 0.001 by two-tailed student’s *t*-test.

To validate the results from iPSC models, we analyzed synaptosomes isolated from neuron-specific ABCA7 knockout mice (n*Abca7*^−^^/^^−^). *Abca7*^floxp/floxp^ mice (Fig. 6A) were crossed with Camk2a-Cre^+/^^−^ mice to generate *Abca7*^floxp/floxp^; Camk2a-Cre^+/^^−^ (n*Abca7*^−^^/^^−^) mice and *Abca7*^floxp/floxp^ (control) mice. Neuron-specific deletion of ABCA7 was confirmed by immunostaining in the hippocampal sections (Fig. 6B) and Western blotting in the extracted synaptosomes from n*Abca7*^−/−^ mice (Fig. 6C). Amounts of mitochondria-related proteins including TIM23, MFN2, DRP1, and FIS1 did not significantly differ between control and n*Abca7*^−/−^ synaptosomes from mice at 4 months of age (Supplementary Fig. 8A). We observed significant reduction of inner mitochondrial membrane protein TIM23, but not MFN2, DRP1, and FIS1, in synaptosomes from n*Abca7*^−/−^ mice compared to those from controls at the age of 20 months (Supplementary Fig. 8C). Consistent with the results from the iPSC models, electron microscopy revealed altered mitochondrial morphology within the extracted synaptosomes in n*Abca7*^−/−^ mice at 20 months of age. The length, width, area, and perimeter of mitochondria were significantly larger in the synaptosomes isolated from n*Abca7*^−/−^ mice than those from control mice (Fig. 6D, E). We also found the reductions of synaptic proteins SNAP25 and PSD95 in synaptosomes from n*Abca7*^−/−^ mice at 20 months age (Fig. 6F), whereas there was no difference at 4 months of age (Supplementary Fig. 8B). Furthermore, mRNA expression levels of these synaptic genes were reduced in cortex from n*Abca7*^−/−^ mice at the age of 20 months (Fig. 6G). Taken together, these results confirm that neuronal ABCA7 deficiency causes synaptic mitochondrial morphology alterations and synaptic protein reduction in mouse brains as well as human iPSC models. We did not observe significant differences in the mRNA levels of *Nnat*, *Rimbp2*, *Iah1*, and *Dnaja4* in the cortex between control and n*Abca7*^−/−^ mice at 20 months (Supplementary Fig. 8D), although these genes were identified as DEGs between control and *ABCA7*^−/−^ iPSC-derived cortical organoids.

## Discussion

Mitochondria are central organelles in maintaining cellular homeostasis as they are vital in energy production, metabolism, intracellular signaling, and apoptosis [38]. Mitochondrial dysfunctions, such as compromised ATP production, excessive ROS generation, and disturbed mitochondrial dynamics have been shown to substantially contribute to AD pathogenesis [43]. While loss-of-function variants in *ABCA7* are associated with increased AD risk, for the first time, we demonstrated that ABCA7 deficiency leads to neuronal mitochondria dysfunctions and diminished synaptic activity using iPSC-derived neuronal models and neuron-specific *Abca7* knockout mice.

ABCA7 is mainly localized in plasma membranes, endoplasmic reticulum (ER), and Golgi apparatus [44, 45], where it plays a critical role in in maintaining cellular lipid homeostasis by mediating lipid transport across organelle membranes [10]. Our lipidomics analysis found that mitochondria-rich phospholipids, particularly PG, were decreased in *ABCA7*^−/−^ iPSC-derived cortical organoids compared to the isogenic controls. ABCA7 deficiency also modulated amounts of some CL subgroups in the organoids, which were identified as hub lipids influencing the lipid profile in the lipidomics. However, PA was increased in *ABCA7*^−/−^ iPSC-derived cortical organoids. PA is synthesized in ER as the precursor for other phospholipids, some of which are converted to PG and then CL in mitochondria [46]. When NBD-labeled PA was exogenously added, we found that PA accumulation was increased in ER of *ABCA7*^−/−^ iPSC-derived neurons. Since transferring lipids between the ER and mitochondria is one of the main processes in phospholipid synthesis [46, 47], our findings suggest that ABCA7 deficiency in ER impairs PA translocation from ER to mitochondria, resulting in excess intracellular accumulation of PA and reduction of PG synthesis. Mitochondrial lipid components also substantially influence mitochondrial dynamics and morphology. CL promotes both Opa1-mediated mitochondrial fusion in the inner membrane and Drp1-mediated mitochondrial division in the outer membrane [38]. PA and CL interact with the core components of mitochondrial fusion and division [38]. Therefore, maintaining homeostasis of mitochondrial lipids, such as PA, PG, and CL, is likely essential for regulated mitochondrial dynamics. Altered lipid metabolism may lead to the enlarged mitochondria morphology observed in ABCA7-deficient iPSC-derived cortical organoids and neurons, as well as synaptosomes from neuron-specific *Abca7* knockout mice. In addition to morphological changes in mitochondria, ABCA7 deficiency also disturbs mitochondria respiration, suppresses ATP production, and increases ROS generation in the iPSC-derived cortical organoids and neurons. Indeed, altered mitochondrial fusion or fission have been known to influence not only mitochondria morphology, but also mitochondrial functions [48].

Since neurotransmission is a highly energy-demanding process, neuronal activity substantially depends on mitochondrial oxidative metabolism [49, 50]. Thus, dysfunction of synaptic mitochondria leads to neuronal apoptosis and consequently impairs neurotransmission and synaptic plasticity [51]. Consistently, our results demonstrated that ABCA7 deficiency disturbs synaptic firing and network formation in iPSC-derived neurons. Importantly, PG administration rescues synaptic function and mitochondrial respiration in *ABCA7*^−/−^ iPSC-derived neurons. Thus, disturbances of mitochondria lipid homeostasis due to ABCA7 deficiency may be one of the mechanisms driving mitochondrial and synaptic dysregulation in neurons. Furthermore, NMN also improved mitochondrial respiration and had notable long-term effects in rescuing synaptic functions of *ABCA7*^−/−^ iPSC-derived neurons. Because NAD^+^, converted from NMN, is an essential energy substrate in mitochondria metabolism [42, 52], the synaptic dysregulation of *ABCA7*^−/−^ iPSC-derived neurons may be the consequent of disturbances in mitochondrial respiration. Indeed, low levels of ATP synthase [53] and NAD^+^ [54] were detected in AD brains. Furthermore, several studies have illustrated the therapeutic effects of NMN in improving synaptic functions and cognitive performance in amyloid model mice [55–57]. Since mitochondrial respirations were not disturbed by ABCA7 deficiency at iPSC and NPC stages, the effect of ABCA7 deficiency on mitochondria may become evident in cell types which require abundant mitochondrial metabolism. In addition, ABCA7 loss-of-function may synergically cause mitochondria damage with aging or other cellular stresses in AD. Future studies should define how ABCA7 loss-of-function impacts mitochondria properties in other brain cell types, and in other physiological and pathological conditions depending on sex.

In summary, our study suggests a novel mechanism by which ABCA7 loss-of-function causes AD-related neuronal impairments. ABCA7 deficiency disturbs mitochondria lipid metabolism, leading to disruption of mitochondrial homeostasis and synaptic function in neurons. Several genetic studies for AD have identified loci clustering mainly in the pathways related to lipid metabolism, immune response, and intracellular trafficking [5, 58]. While lipid metabolism is critically involved in immune response and membrane trafficking, we provide further evidence that lipid dysregulation drives mitochondrial and synaptic dysfunctions. In addition, we also show that recovering mitochondrial function with PG supplementations can ameliorate synaptic dysregulation in *ABCA7*^−/−^ iPSC-derived neurons. Our results indicate that mitochondrial lipids may be new therapeutic targets to treat neuronal dysfunctions in AD.

### Supplementary information

Supplemental material

Data set

## Data Availability

All data generated for this manuscript have been included in this article. The bulk RNA-seq data that support the findings of this study are deposited in the Gene Expression Omnibus repository under accession number GSE247360. All other data are available from the corresponding author upon reasonable request.
