# Supplementary material for: ABCA7 deficiency causes neuronal dysregulation by altering mitochondrial lipid metabolism
Source: Mol Psychiatry. 2023 Dec 22;29(3):809–19. doi: 10.1038/s41380-023-02372-w (PMC11153016; doi:10.1038/s41380-023-02372-w)
Supplement: Supplementary file 1 — Supplemental material [file 41380_2023_2372_MOESM1_ESM.pdf]

Supplementary Table 1. Primer information used in qRT-PCR

Species	Gene	Forward primer (5'-3')	Reverse primer (5'-3')
Human	*GAPDH*	GTCTCCTCTGACTTCAACAGCG	ACCACCCTGTTGCTGTAGCCAA
	ABCA7	CCTTGCACAGCTTGTTGGAG	CTGCTAGGTCCCCTGACACT
	NNAT	GTGTTCCTGGAATGCTGCATTTAC	GACACCGTGTATGCCAGCTTCT
	RIMBP4	AGGACGCCTATGACTCTCCAGA	CTCTGTGTGGTACTCGTCTCCA
	IAH1	TCCGTGGACATCCCTGAGAATC	CCACTTGTAAACACGCATTGGCA
	DNAJA4	GAATGCCCATCTACAAAGCACCC	TCTGTCGAGGAGGGAGTAAAGC
	APAF1	GCCAAGCAGGAGGTCGATAATG	GACCATCCTCAGAAAAGCAGGC
	BAK1	TTACCGCCATCAGCAGGAACAG	GGAACTCTGAGTCATAGCGTCG
	XIAP	TGGCAGATTATGAAGCACGGATC	AGTTAGCCCTCCTCCACAGTGA
	ND6	Hs02596879 (Thermo Fisher Scientific)	
	CYB	Hs02596867 (Thermo Fisher Scientific)	
	CO1	Hs02596864 (Thermo Fisher Scientific)	
	CO3	Hs02596866 (Thermo Fisher Scientific)	
	ATP6	Hs02596862 (Thermo Fisher Scientific)	
	MFN1	Hs00966851 (Thermo Fisher Scientific)	
	MFN2	Hs00208382 (Thermo Fisher Scientific)	
	MFF	Hs00697394 (Thermo Fisher Scientific)	
	FIS1	Hs00211420 (Thermo Fisher Scientific)	
	OPA1	Hs01047019 (Thermo Fisher Scientific)	
	DNML	Hs01552605 (Thermo Fisher Scientific)	
	GAPDH	Hs02786624 (Thermo Fisher Scientific)	
Mouse	*Snap25*	CTGGCTGATGAGTCCCTGGAAAGCACCC	TCCCGGGCATCATTTGTTACCCTGCGG
	Dlg4	GACGCCAGCGACGAAGAG	CTCGACCCGCCGTTTG
	Nnat	GTGGTGGAGGAAGAGGGTTAAG	CACATTTTGGGGAGGGCTTTCG
	Rimbp2	GAGCAGAGAGAAAAGAAGGAGGC	CTTCCAGGAGACCTGAACAGAG
	Iah1	TGGACCCTGATGCAGAAGGACA	GATGAGACCTTCTTGTCCAGCAG
	Dnaja4	ATCGGAAGCTGGCGCTCAAGTA	TGCCTGTTCTCCACCTTGGTCA
	Gapdh	TGCCCCCATGTTTGTGATG	TGTGGTCATGAGCCCTTCC

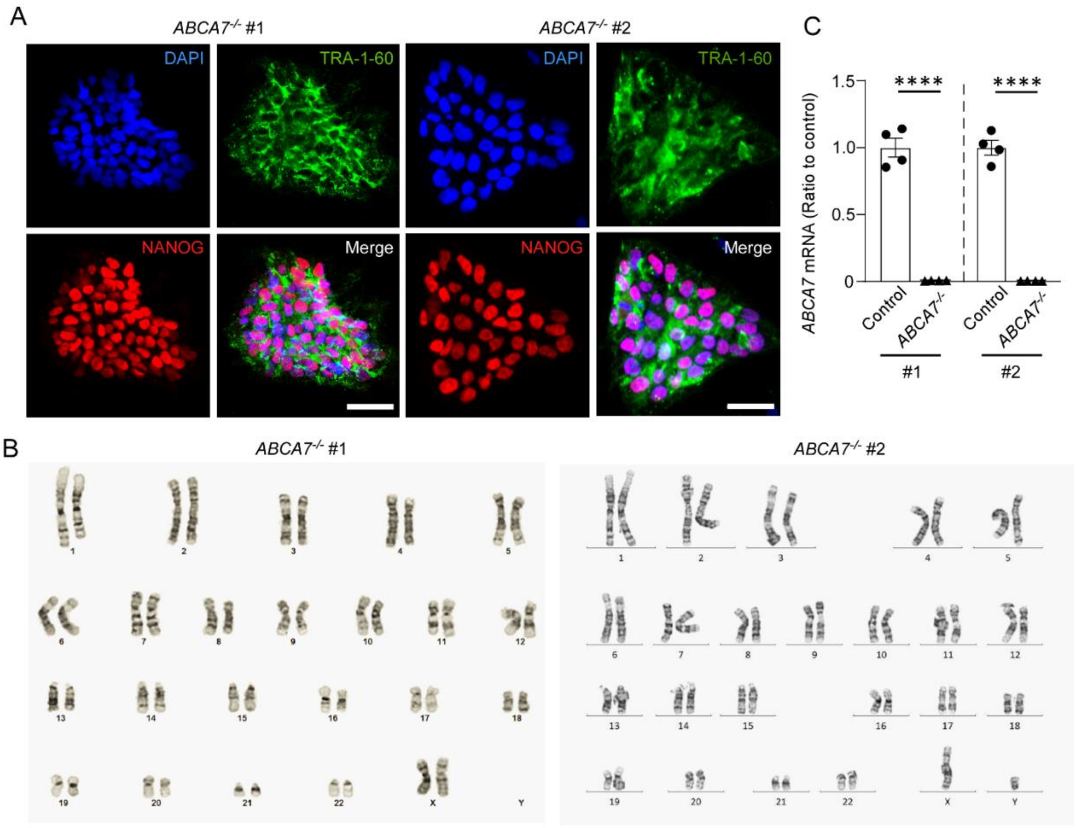

Supplementary Figure 1. Characterization of *ABCA7*$^{-/-}$ iPSC lines. **(A)** Immunocytochemical staining for pluripotency markers using specific antibodies against NANOG and TRA-1-60 in *ABCA7*$^{-/-}$ iPSC lines (#1 and #2). Nuclei were stained with DAPI. Scale bars: 50 µm. **(B)** Karyotyping in *ABCA7*$^{-/-}$ iPSC lines (#1 and #2). **(C)** Expression of *ABCA7* mRNA in the iPSC lines (#1 and #2) was measured by qRT-PCR and normalized by *GAPDH* mRNA level (n = 4 technical replicates/line). Data represents mean ± SEM. ****$p < 0.0001$ by two-tailed student's *t*-test.

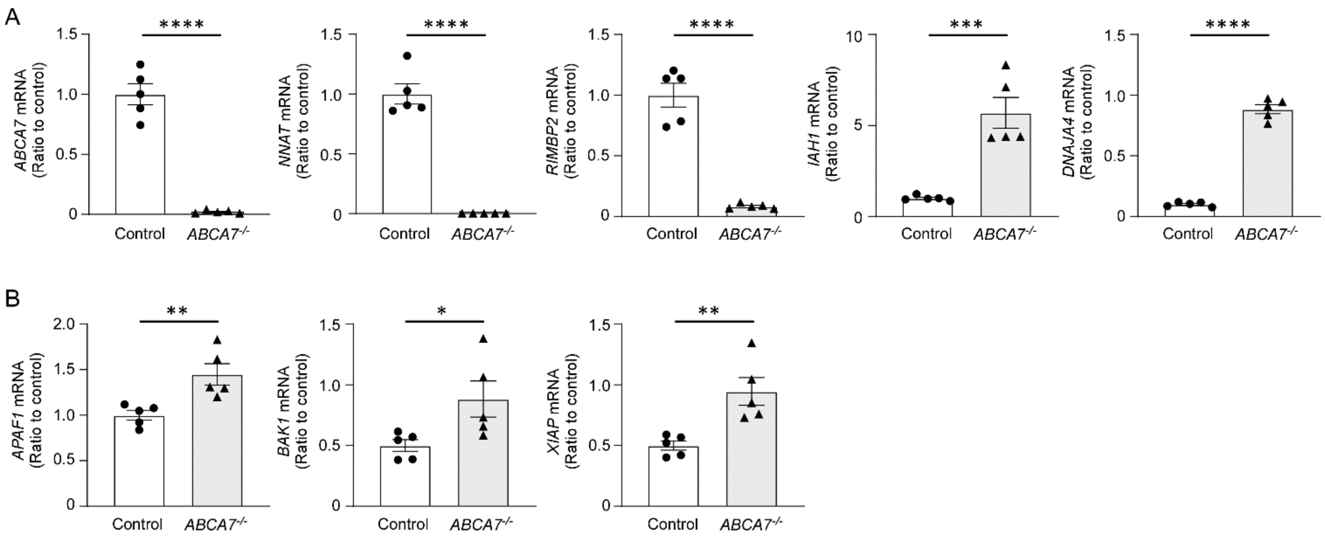

Supplementary Figure 2. Validation of RNA-seq results using qRT-PCR in iPSC-derived cortical organoids. The mRNA levels of RNA-seq identified DEGs (A) and apoptosis marker genes (B) in isogenic control and *ABCA7*$^{-/-}$ iPSC-derived cortical organoids were measured by qRT-PCR, followed by normalization with *GAPDH* mRNA level (n = 5 technical replicates /line). Data represents mean ± SEM. *p < 0.05, **p < 0.01, ***p < 0.001, ****p < 0.0001 by two-tailed student's *t*-test.

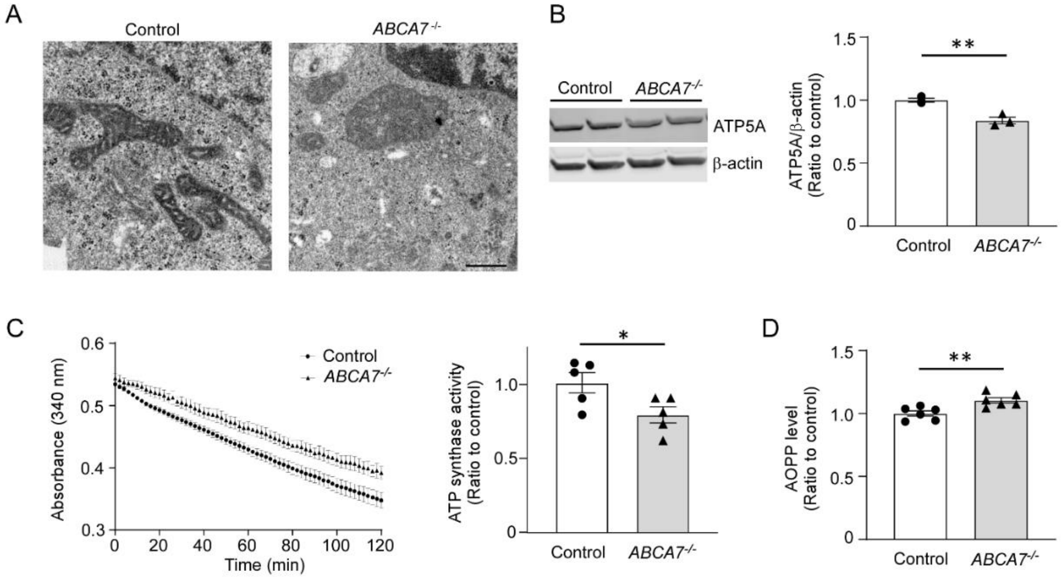

Supplementary Figure 3. Altered mitochondrial properties in *ABCA7*$^{-/-}$ iPSC-derived cortical organoids. (A) Representative electron microscope images of mitochondria in the cortical organoids derived from isogenic control and *ABCA7*$^{-/-}$ iPSCs (#1) are shown. Scale bar: 500 nm. (B) ATP5A (component V) levels in the iPSC-derived cortical organoids were analyzed by Western blotting and normalized by β-actin levels (n = 3 technical replicates/line). (C) ATP synthase enzyme activity in the iPSC organoids was monitored for 120 minutes through ATP synthase Enzyme Activity Microplate Assay Kit. ATP synthase enzyme activity for 20 min interval between 40 to 60 minutes were plotted (n = 5 technical replicates/line). (D) Amounts of advanced oxidation protein product (AOPP) in the iPSC organoids were measured through AOPP Assay Kit (n = 6 technical replicates/line). Data represents mean ± SEM. $^{*}p < 0.05$, $^{**}p < 0.01$ by two-tailed student's *t*-test.

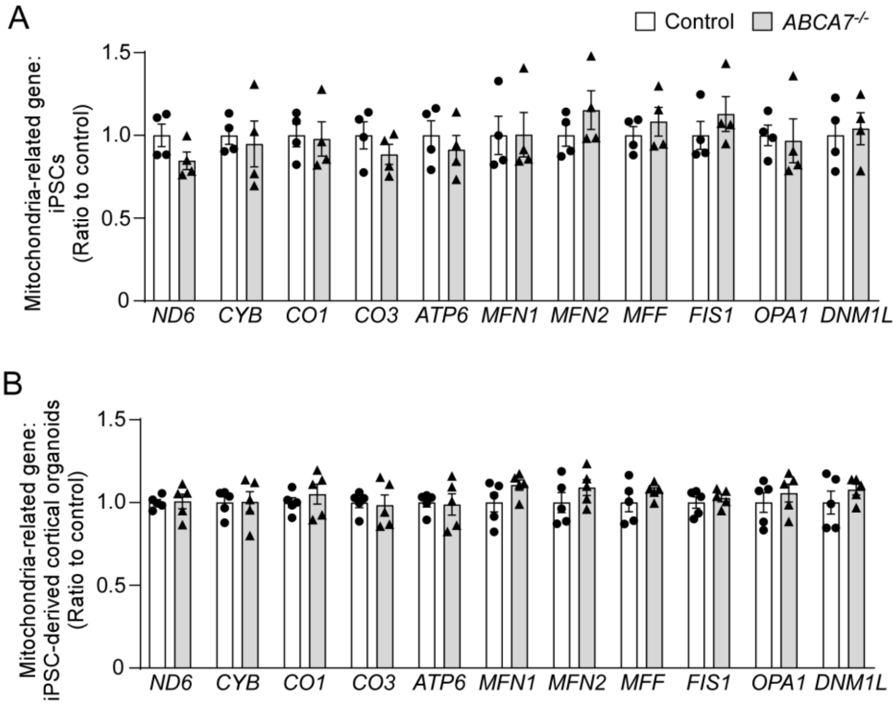

Supplementary Figure 4. Expression of mitochondria-related genes in *ABCA7*$^{-/-}$ iPSCs and the iPSC-derived cortical organoids. Expression levels of mitochondria-related genes including mitochondrial mRNAs (*ND6*, *CYB*, *CO1*, *CO3*, and *ATP6*) and nuclear mRNAs (*MFN1*, *MFN2*, *MFF*, *FIS1*, *OPA1*, and *DNM1L*) in isogenic control and *ABCA7*$^{-/-}$ iPSC #1 (A) and the iPSC-derived cortical organoids (B) were measured by qRT-PCR, followed by normalization with *GAPDH* mRNA level (n = 4–5 technical replicates /line). Data represents mean ± SEM. Not significant ($p > 0.05$) by two-tailed student's t-test.

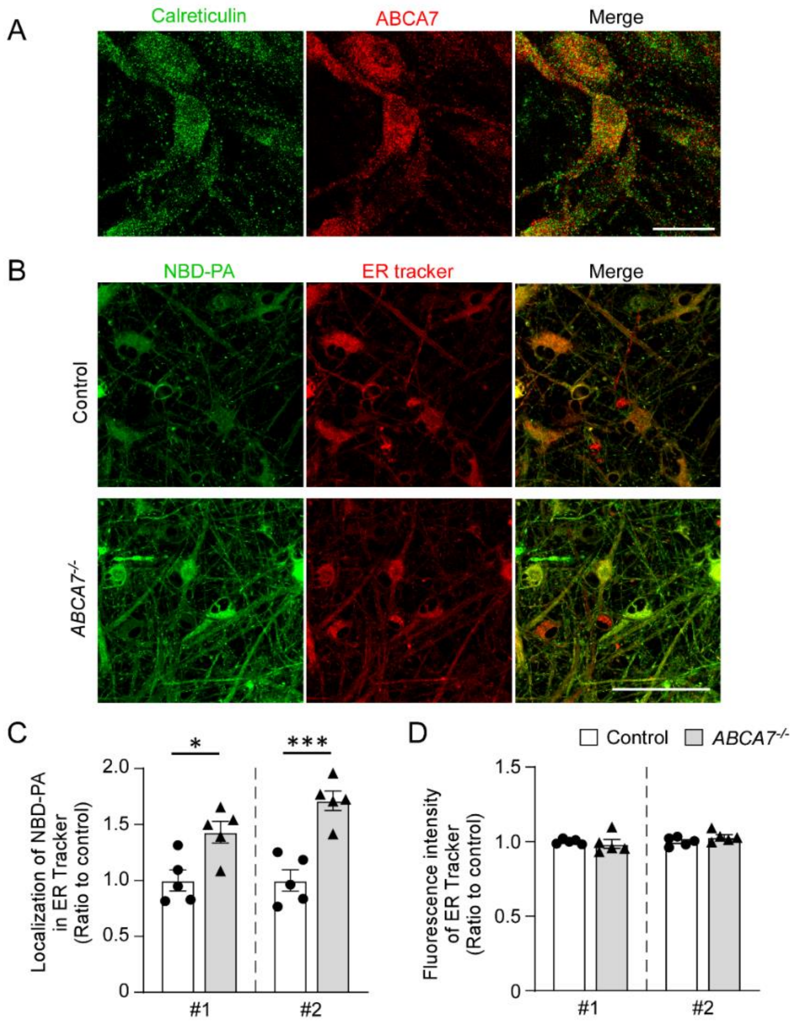

Supplementary Figure 5. Increased accumulation of NBD-labeled phosphatidic acid in ER of *ABCA7*$^{-/-}$ iPSC-derived neurons. (A) Immunocytochemical staining for ABCA7 and ER marker Calreticulin in iPSC-derived neurons. Scale bar: 25 µm. (B) Confocal imaging analysis of iPSC-derived neurons (#1 and #2) with NBD-PA_16:0-06:0 and ER tracker. (C) Mean intensity of ER tracker (n = 5 technical replicates/line). (D) Mean intensity of NBD-PA merged ER tracker (n = 5 technical replicates/line). Data represents mean ± SEM. *p < 0.05, ***p < 0.001 by two-tailed student's *t*-test.

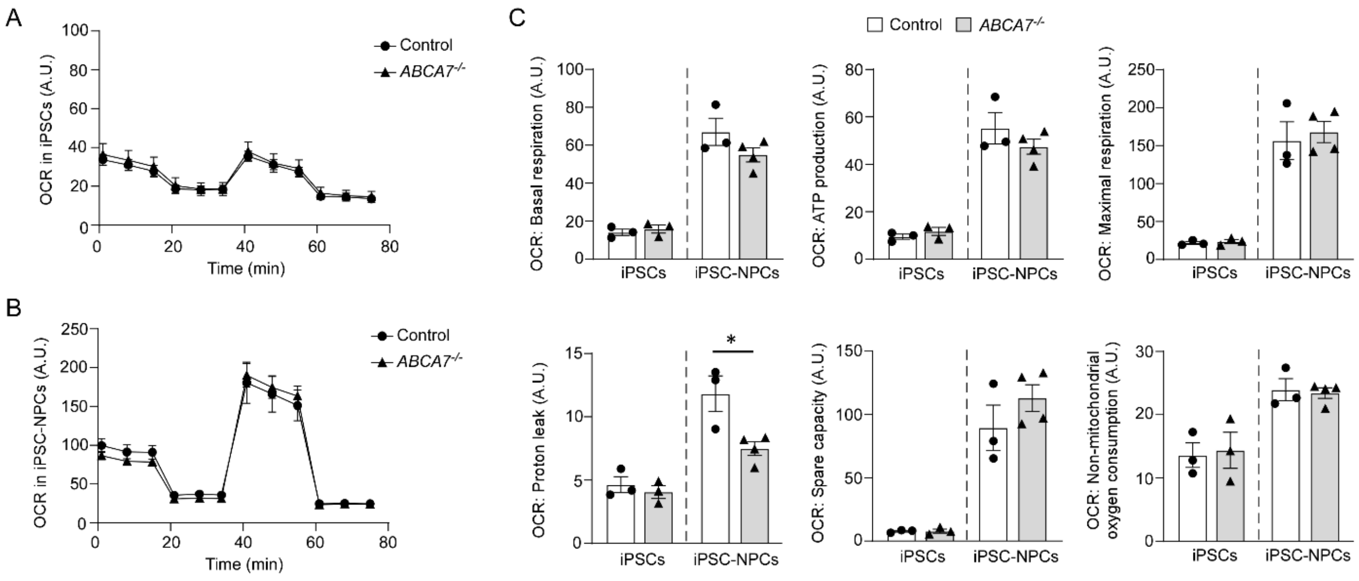

Supplementary Figure 6. Mitochondrial respiration in *ABCA7$^{-/-}$* iPSCs and the iPSC-derived NPCs. Mitochondrial respiration in isogenic control and *ABCA7$^{-/-}$* iPSCs (#1) (A and C) and the iPSC-derived NPCs (B and C) was measured by Mito Stress Test Kit through Seahorse XFe96 Extracellular Flux Analyzer (n = 3–4 technical replicates/line). The OCR measurements were normalized to cell density determined by nuclear DAPI staining in each well. A.U., arbitrary unit. Data represents mean ± SEM. *$p < 0.05$ by two-tailed student's *t*-test.

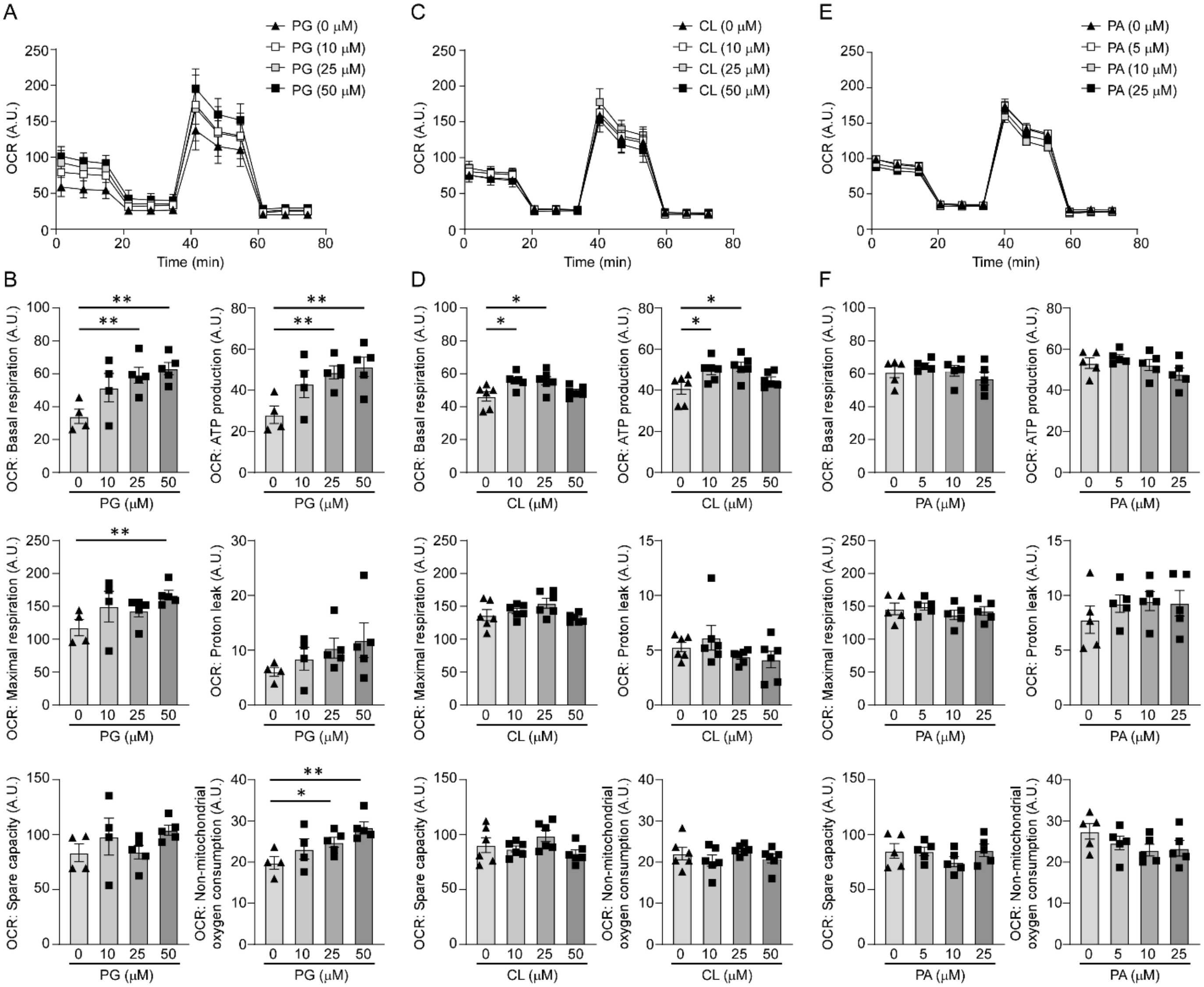

Supplementary Figure 7. Effects of administrations with PG, CL, and PA on mitochondrial respiration in *ABCA7*$^{-/-}$ iPSC-derived neurons. *ABCA7*$^{-/-}$ iPSC-derived neurons (#1) were treated with different concentration of PG (A, B), CL (C, D), or CL (E, F) for 1 day 7 weeks after the differentiation from NPCs and mitochondrial respiration was measured by Mito Stress Test Kit through Seahorse XFe96 Extracellular Flux Analyzer (n = 4–6 technical replicates/group). A.U., arbitrary unit. Data represents mean ± SEM. *$p < 0.05$, **$p < 0.01$ by two-tailed student's t-test.

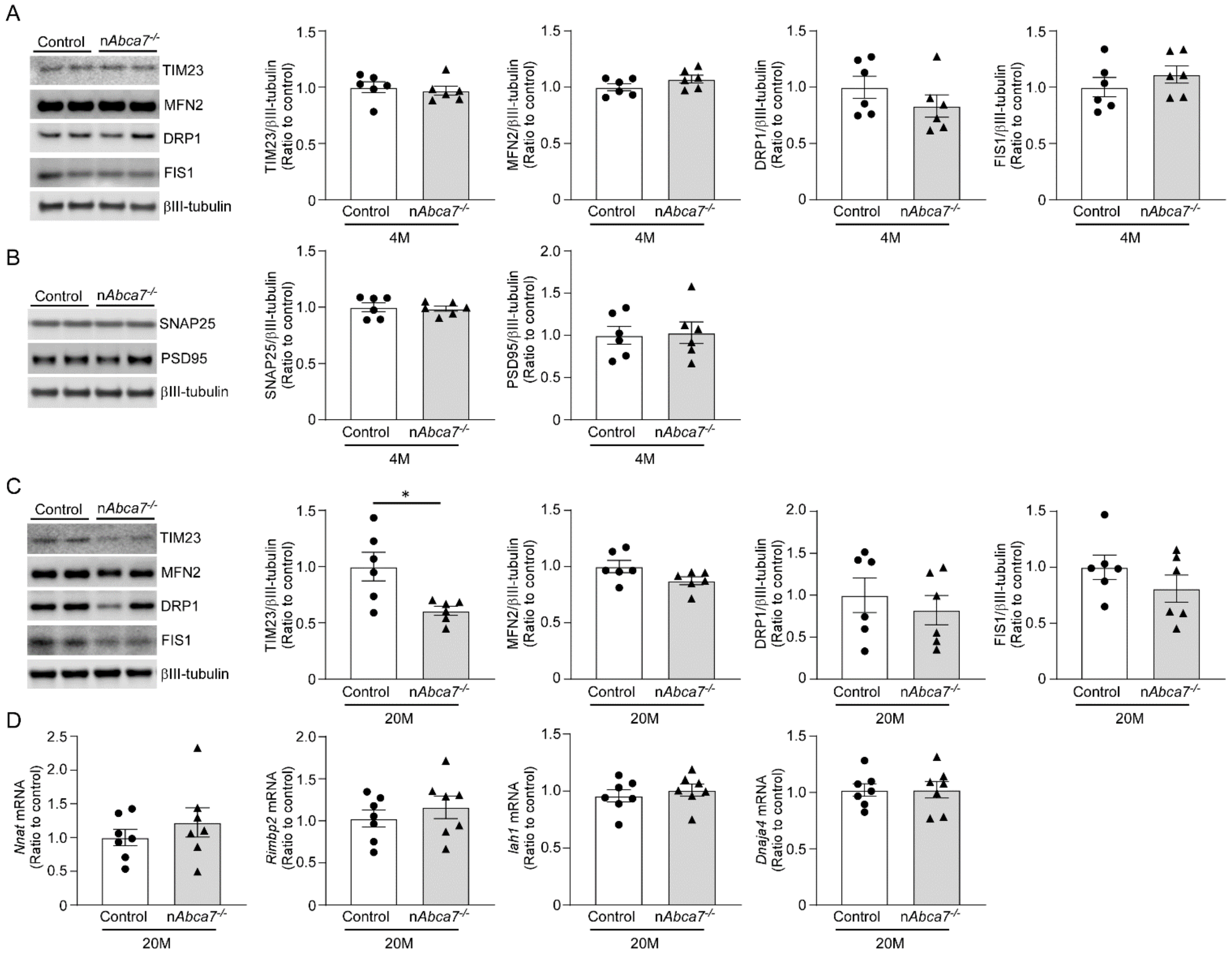

Supplementary Figure 8. Effects of neuronal ABCA7 deficiency on mitochondria and synaptic protein levels in mouse synaptosomes. Mitochondria-related protein levels (A) and synaptic protein levels (B) were measured by Western blotting of 4-month-old mouse synaptosomes (N = 6 male mice/group). (C) Mitochondria-related protein levels were measured by Western blotting in the synaptosomes extracted from mouse brains at the age of 20 months (N = 6 male mice/group). Data were normalized to βIII-tubulin (N = 6 male mice/group). (D) The mRNA levels of *Nnat*, *Rimbp2*, *Iah1*, and *Dnaja4* were measured by qRT-PCR and normalized by *Gapdh* mRNA level (N = 7 male mice/group). Data represents mean ± SEM. *p < 0.05 by two-tailed student's t-test.
